# The UK Out of Hospital Cardiac Arrest Outcome (OHCAO) project

**DOI:** 10.1136/bmjopen-2015-008736

**Published:** 2015-10-01

**Authors:** Gavin D Perkins, Samantha J Brace-McDonnell

**Affiliations:** Warwick Clinical Trials Unit, Division of Health Sciences, Warwick Medical School, The University of Warwick, Coventry, UK

**Keywords:** EPIDEMIOLOGY, ACCIDENT & EMERGENCY MEDICINE, STATISTICS & RESEARCH METHODS

## Abstract

**Introduction:**

Reducing premature death is a key priority for the UK National Health Service (NHS). NHS Ambulance services treat approximately 30 000 cases of suspected cardiac arrest each year but survival rates vary. The British Heart Foundation and Resuscitation Council (UK) have funded a structured research programme—the Out of Hospital Cardiac Arrest Outcomes (OHCAO) programme. The aim of the project is to establish the epidemiology and outcome of OHCA, explore sources of variation in outcome and establish the feasibility of setting up a national OHCA registry.

**Methods and analysis:**

This is a prospective observational study set in UK NHS Ambulance Services. The target population will be adults and children sustaining an OHCA who are attended by an NHS ambulance emergency response and where resuscitation is attempted. The data collected will be characterised broadly as system characteristics, emergency medical services (EMS) dispatch characteristics, patient characteristics and EMS process variables. The main outcome variables of interest will be return of spontaneous circulation and medium—long-term survival (30 days to 10-year survival).

**Ethics and dissemination:**

Ethics committee permissions were gained and the study also has received approval from the Confidentiality Advisory Group Ethics and Confidentiality committee which provides authorisation to lawfully hold identifiable data on patients without their consent. To identify the key characteristics contributing to better outcomes in some ambulance services, reliable and reproducible systems need to be established for collecting data on OHCA in the UK. Reports generated from the registry will focus on data completeness, timeliness and quality. Subsequent reports will summarise demographic, patient, process and outcome variables with aim of improving patient care through focus quality improvement initiatives.

**Strengths and limitations of this study**

- Successful accomplishment of objectives highly likely to improve understanding and improve outcomes from UK population, and potential to influence national policy and procedures.
- This is a unique opportunity to study the impact of ‘process’ on national patient outcomes.
- The development of operational procedures, standardised data collection processes and data definitions.
- Reliance on already stretched National Health Service (NHS) resources.

## Introduction

Reducing premature death is a key priority for the National Health Service (NHS).^1^ ^2^ NHS Ambulance Services treat approximately 30 000 patients a year for out of hospital cardiac arrest. There is significant variability between ambulance services in rates of the reported successful initial resuscitation (13–27%) and survival to hospital discharge (2–12%).^3^ Nichol *et al* identified evidence of regional variation in incidence and outcomes from OHCA in 10 North American sites. There was more than 100% variability in incidence (rates ranging from 71 to 160/100 000 population) and similar variability in the decision to start resuscitation. Of those patients where resuscitation was started by the emergency medical service (EMS) there was marked variation in survival rates (range 3.0–16.3%, with a median of 8.4% (IQR, 5.4–10.4%).^4^

**Figure 1:**
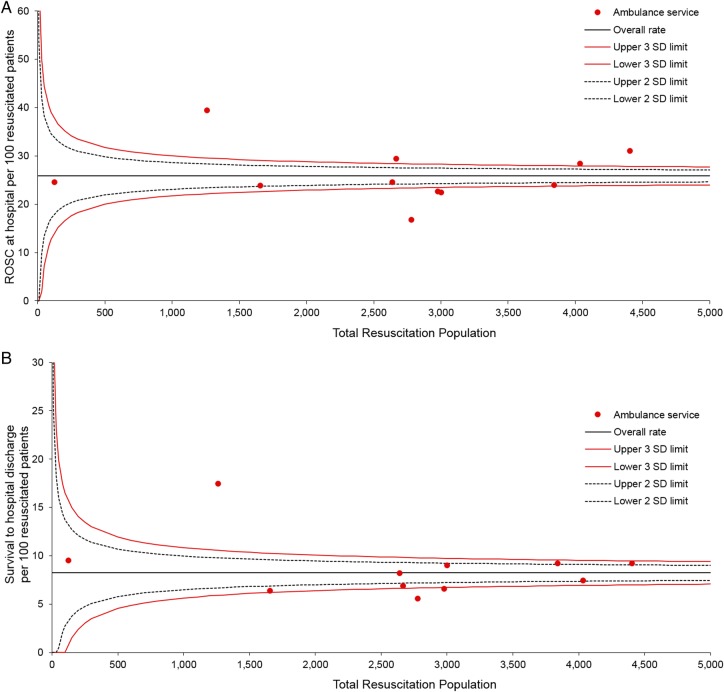
Funnel plot showing percentage return of spontaneous circulation (A) and survival to hospital discharge (B) against the total number of cardiac arrests where resuscitation was attempted. Each dot represents a single ambulance service. Variation within the dotted line boundaries are considered to be due to normal or common-cause variation. Those lying outside the dotted line represent cases of special cause variation.

Differences in outcomes may occur due to random variation (so called common-cause variation) or due to non-random/special cause variation. The former is to be expected in any process or system, while the latter is a systematic or unexpected deviation from the norm and may highlight an area worthy of further investigation. Evaluation of the English ambulance services return of spontaneous circulation (ROSC) and survival to discharge rates suggests there may be special cause variation (see figure 1).

### Potential explanations for special cause variability

**Figure 2:**
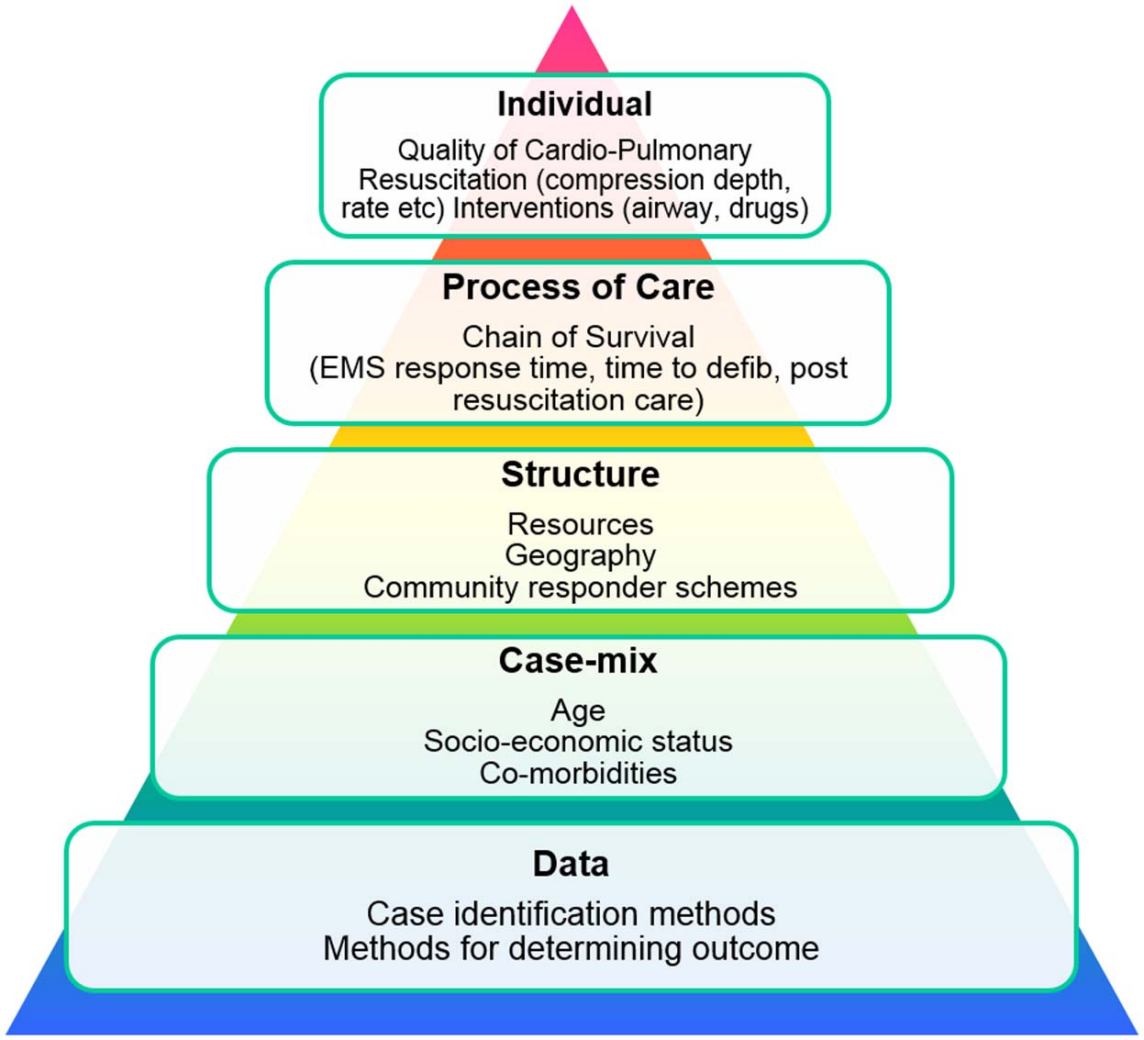
Sources of special cause variation in cardiac arrest likely to influence survival rates adapted from Lilford *et al*.^5^ EMS, emergency medical services.

Lilford *et al*^5^ describes a pyramid with five causes of non-random/special variation in health outcomes (data, case mix, structure, process of care, individual). The concept behind the pyramid is that most variation arises from inconsistencies in data (hence the base of the pyramid) reducing to individual practitioner variation as the smallest contributor. Figure 2 shows these principles applied in the context of cardiac arrest.

Differences in data collection processes can have a dramatic impact on reported outcomes. Since the rate of cardiac arrest survival is derived from the number of people who survive divided by the number of resuscitation attempts, consistency with the processes used to determine the number of cases (case ascertainment) and outcome verification is critical for ensuring systems compare like with like. Early exploratory work in the UK has identified five different ways through which ambulance services identify cases of cardiac arrest.^6^ Each approach may identify patients with subtly different characteristics and outcomes. For example, cases identified by EMS dispatch systems as cardiac arrest have a higher rate of survival (due to telephone CPR instructions and more rapid EMS response) than cases missed. Reliance solely on EMS dispatch codes to identify cardiac arrest cases would inflate survival rates relative to systems that included cases which were missed by dispatchers. Differences in case ascertainment processes might explain the observed variation in the proportion of category A Red 1 999 calls (life threatening emergency) reported as cardiac arrest cases.

The Utstein templates^7^ ^8^ aim to provide consistency to the data definitions used by cardiac arrest registries. However, it is important that definitions are consistently applied to reduce variation.^9^

Differences in the case mix of patients attended by ambulance services, for example, age,^10^ ^11^ sex,^12^ body mass index,^13^ race,^14^ social deprivation^14^ ^15^ are known to influence outcome. The Utstein comparator group (bystander witnessed cardiac arrest who are in VF) attempts to allow some adjustment for case mix, although it likely accounts for only 40% of the observed variation.^16^ More complex statistical adjustments for case mix may be helpful in reducing variation due to differences in case mix.^17^ Structural factors may include geography,^18^ the provision and uptake of public access defibrillation,^19^ ^20^ community initiatives.^21^ Process variables include EMS response time,^22^ time to first shock and likely the facilities at the receiving hospital.^23^ Variation attributable to the individual care provider most likely relates to the quality of CPR^24^ and thresholds for initiating resuscitation.

## Aims and objectives of the project

The aim of the project is to (1) establish the epidemiology and outcome of OHCA, (2) explore sources of variation in outcome and (3) establish the feasibility of setting up a national OHCA registry as a quality improvement and research tool.

## Methods/design

This is a prospective observational study set in UK NHS ambulance services. UK ambulance services serve a population of 63 270 000 people.^25^ Each ambulance service operates at least one emergency operations centre which coordinates all ambulance activity. In 2013 UK ambulance services received 9.1 million 999 calls, 7 million of these required an emergency response and of these 2.7 million were classified as needing an 8 min response. These calls generated 5 million journeys to emergency departments, of which there are approximately 247 emergency departments in the UK.^26^ ^27^ Clinical treatment protocols follow guidelines from the Association of Ambulance Chief Executive,^28^ Intensive Care Society^29^ and Resuscitation Council (UK).^30^

The target population for the project will be adults and children sustaining an OHCA who are attended by an NHS ambulance emergency response and resuscitation is attempted. The data collected will broadly be characterised as system characteristics, EMS dispatch characteristics, patient characteristics and EMS process variables. The main outcome variables of interest will be ROSC and medium—long-term survival (30 days to 10-year survival). The project will work to standardise definitions used across ambulance services and to align them with the Utstein recommendations for Out of Hospital Cardiac Arrest.^8^ See table 1 for the collected variables.

**Table 1:** Summary of data options and definitions for the OHCAO project

Class	OHCAO long name	OHCAO consensus definition
System *(Annual statement)*	Population served *(Core)*	Total current population living within service area of EMS system. Boarders defined by CCG areas served by that EMS service and population living within as stated by the Office of National Statistics for the latest year available Population defined by Local Health Board boarder where CCG’s do not operate
	Number of cardiac arrests attended *(Core)*	Number of cardiac arrests attended (arrests defined by absence of signs of circulation) Inclusion criteria: If EMS response started/continued ALS or BLS ROLE completed Successful resuscitation by a Bystander before an EMS response arrives Exclusion criteria: If bystander CPR started and not continued by EMS Valid DNAR order is in place Valid advanced refusal of treatment is in place
	Resuscitation attempted *(Core)*	When EMS personnel perform chest compressions or attempt defibrillation, it is recorded as a resuscitation attempt by EMS personnel
	Resuscitation not attempted *(Core)*	Total number of cardiac arrests in which resuscitation was not attempted and the number of those arrests not attempted because a written DNACPR order was present or victim was obviously dead or signs of circulation were present
	System description *(Core)*	A description of the organisational structure of the EMS service being provided. This should encompass the levels of service delivery, annual case numbers, and size of geographic region covered
	System description *(Supplemental)*	System information: Free text description defining (A) the presence or existence of legislation that mandates no resuscitation should be started by EMS or health services in specific circumstances or clinical cohorts of patients; (B) systems for limiting/terminating prehospital resuscitation; (C) termination of resuscitation rules; (D) whether dispatch software is used (and type, version); (E) resuscitation algorithms followed (eg, AHA, ERC, any local variations, CPR or shock first, compression-only CPR initially/compressions and ventilations). (F) Describe any formalised data quality activities in place. (G) Describe prehospital ECG capability: if EMS system has ability to perform and interpret (or have interpreted via telemetry) 12-lead ECGs in the field
Patient sensitive data	Patient’s surname *(Core)*	
	Patient’s forename *(Core)*	
	Patient’s NHS number *(Core)*	The unique identifier for a patient within the NHS in England or Wales, or the Scottish Community Health Index number
	Patient’s general practitioner or surgery identifier *(Supplemental)*	*Note:* National code which identifies the GP practice if available
	Patient’'s full home address *(Core)*	
	Patient’s home postcode *(Core)*	*Note:* OHCAO project will overwrite any details found from data linkage
Patient	Type of PRF *(Core)*	
	PRF serial number *(Core)*	
	Regional ambulance incident case number *(Core)*	*Note: if multiple patients, please add a sequential letter*
	Patient’s date of birth *(Core)*	If the victim's date of birth is known, it should be recorded in an acceptable format. If the date of birth is not known but the victim's age is known, age should be recorded. If the victim's age is not known, age should be estimated and recorded
	Patient’s age *(Core)*	
	Age unit *(Core)*	*Note: ‘*Blank’ values will be assumed as ‘1 Years’
	Patient’s sex *(Core)*	Sex of the patient at birth *Note: ‘*Blank’ values will be assumed as ‘99 Unknown’
	Patient’s ethnicity *(Core)*	List as provided by NHS England *Note:* ‘Blank’ entries will be assumed as ‘Z not Stated’
Event	Date of emergency medical services call *(Core)*	Date of receipt of dispatch call
	Time of emergency medical services call—‘Call Connect time’ *(Core)*	The time that the call is connected to the ambulance service by the BT operator
	Response times *(Core)*	The time interval from ‘Call Connect time’ to the time the first organised ‘emergency medical service response vehicle’s wheel stops on scene’ at a point closest to the patient's location. Organised EMS response includes CFR’s sent *Note:* If ‘Call connect time’ is not available, then ‘Inc Clock Start’ time may be used *Note:* CAD clock stops at 200 m of actual vehicle stop time. AS to check which is available *Note:* If ‘Blank’, will be calculated from ‘Time of Emergency Medical Services call’ and ‘Time Emergency Medical Services vehicle stops’
	Computer-aided dispatch classification *(Supplemental)*	NHS pathways categorisation *Note: ‘*Blank’ entries will be assumed as ‘99 Unknown’
	Utstein location of emergency medical services occurrence *(Core)*	The specific location where the event occurred or the patient was found. Knowledge of where cardiac arrests occur may help a community to determine how it can optimise its resources to reduce response intervals. A basic list of predefined locations will facilitate comparisons. Local factors may make creation of subcategories useful *Note:* ‘Blank’ entries will be assumed as ‘Not Recorded’ *Note:* When multiple entries occur, please refer to the ‘Primacy guidance’ that accompanies this document The specific location where the event occurred or the patient was found. Knowledge of where cardiac arrests occur may help a community to determine how it can optimise its resources to reduce response intervals. A basic list of predefined locations will facilitate comparisons. Local factors may make creation of subcategories useful *Note:* ‘Blank’ entries will be assumed as ‘Not Recorded’ *Note:* When multiple entries occur, please refer to the ‘Primacy guidance’ that accompanies this document
Event continued	Full location of emergency medical services occurrence *(Core)*	Location as provided to the EMS responding vehicle
	Post code or map reference location of emergency medical services occurrence (*Supplemental)*	Postcode or map reference as provided to the EMS responding vehicle
	Clinical commissioning group (Local Health Board) *(Supplemental)*	*Note:* Where ‘Blank’, OHCAO project will overwrite from data linkage *Note:* If no CCG is available, then Local Health Board name should be provided
	Occurrence witnessed by? *(Core)*	A cardiac arrest that is seen or heard by another person or is monitored. EMS personnel respond to a medical emergency in an official capacity as part of an organised medical response team. Bystanders are all other groups. By this definition, physicians, nurses or paramedics who witness a cardiac arrest and initiate CPR but are not part of the organised rescue team are characterised as bystanders, and the arrest is not described as EMS witnessed *Note:* When multiple entries occur, please refer to the ‘Primacy guidance’ that accompanies this document
Pre-EMS first aids	Bystander commenced CPR *(Core)*	Bystander CPR is cardiopulmonary resuscitation performed by a person who is not responding as part of an organised emergency response system to a cardiac arrest. Physicians, nurses, and paramedics may be described as performing bystander CPR if they are not part of the emergency response system involved in the victim's resuscitation. Bystander CPR may be compression only (CCCPR) or compression with ventilations (full CPR) (the act of inflating the patient's lungs by rescue breathing with or without a bag-mask device or any other mechanical device) *Note:* When multiple entries occur, please refer to the ‘Primacy guidance’ that accompanies this document
	Public access defibrillator available *(Supplemental)*	According to the CAD system, was there an AED available at the incident location *Note:* OHCAO project will overwrite data from AED event form submission
	Bystander automated external defibrillator (AED) use *(Core)*	Bystander AED use *Note:* OHCAO project will overwrite data from AED event form submission *Note:* When multiple entries occur, please refer to the ‘Primacy guidance’ that accompanies this document
Primary assessments	Was a ROSC noted on arrival of EMS staff? *(Supplemental)*	Occasionally when a bystander witnesses a cardiac arrest and starts CPR, the victim will regain signs of circulation by the time EMS personal arrive. If the bystander verifies that the victim had no signs of circulation and the CPR was performed, a registry record should be initiated, EMS personnel do not need to verify that a cardiac arrest occurred for this case to be included in the registry
	Initial aetiology of cardiac arrest *(Core)*	Includes cases where the cause of the cardiac arrest is presumed to be cardiac, other medical (eg, anaphylaxis, asthma, GI bleed, **Respiratory**), and where there is no obvious cause of the cardiac arrest
	First monitored rhythm *(Core)*	Victim is found submersed in water without an alternative causation
	Do not attempt resuscitation (DNAR) order in place? *(Supplemental)*	A valid DNAR order was in place and observed *Note:* There may be a need for initial treatment to commence whilst a valid DNAR is confirmed and treatment then withdrawn *Note:* If a valid DNAR order is in place, any ‘Blank’ ‘Date of Death’ will be transformed from date of incident
	Emergency medical services chest compressions *(Supplemental)*	Resuscitation (CCCPR or CPR) commenced or continued by EMS either manual or mechanical in an attempt to restore spontaneous circulation *Note:* BLS, ALS, ILS, would all include chest compressions
Primary assessments continued	Continual ventilations given by EMS *(Supplemental)*	EMS provide manual or mechanical ventilations while the patient has made no sustainable respiratory effort *Note:* BLS, ALS, ILS, would all include chest compressions At any time during the resuscitation was a mechanical CPR device deployed?
	Mechanical CPR *(Supplemental)*	*Note:* When multiple entries occur, please refer to the ‘Primacy guidance’ that accompanies this document
	CPR quality monitoring available *(Supplemental)*	During the resuscitation, were there mechanisms or processes in place to measure the quality of CPR being delivered?
	Attempted defibrillation of the patient *(Supplemental)*	Note: If ‘Blank’ than will be transformed from ‘Bystander Automated External Defibrillator (AED) use’, than ‘First monitored rhythm’
	Total number of shocks *(Supplemental)*	The total number of shocks delivered (including shocks delivered by Public Access Defibrillators, Community First Responders and ambulance personnel)
Drug interventions	Vascular access type *(Supplemental)*	The main route through which drugs were administered during the arrest
	Adrenaline *(Core)*	The delivery of the listed medication (by intravenous cannula, intraosseous needle, or tracheal tube) during the resuscitation event *Note:* Volume not required
	Amioderone *(Core)*	Note: If ‘Blank’ than will be transformed from ‘Bystander Automated External Defibrillator (AED) use’, than ‘First monitored rhythm’
	Vasopressin *(Core)*	The delivery of the listed medication (by intravenous cannula, intraosseous needle, or tracheal tube) during the resuscitation event *Note:* Volume not required
	Sodium chloride bolus *(Core)*	
	Glucose/dextrose *(Core)*	
	Naloxone *(Core)*	The main route through which drugs were administered during the arrest
	Drug timings *(Supplemental)*	The time interval from incoming call to the time vascular access is obtained and the first drug is given
Airway management		What was the main prehospital airway management device used?
Outcome	Any ROSC *(Core)*	Did the patient achieve a ROSC at any point during the resuscitation attempt? *Note:* The term ‘any ROSC’ is intended to represent a brief (approximately >30 s) restoration of spontaneous circulation that provides evidence of more than an occasional gasp, occasional fleeting palpable pulse or arterial waveform
	Survived event *(Core)*	Did the patient have ROSC at point of arrival at the emergency department of the receiving hospital?
	12-lead ECG *(Supplemental)*	Was a 12-lead ECG performed after ROSC? At the time of the first 12-lead ECG performed after ROSC, the presence of STEMI is observed
	Presence of STEMI *(Supplemental)*	What was the main prehospital airway management device used?
Outcome continued	Death confirmed by emergency medical services? *(Supplemental)*	ROLE by responded EMS *Note:* If ‘1 Yes’, any ‘Blank’ ‘Date of Death’ will be transformed from date of incident
	Transported to hospital *(Supplemental)*	Was the patient transported to the hospital? *Note:* Overwritten from ‘Receiving hospital code/name’ when ‘Blank’, ‘Unobtainable’ or ‘Unknown’
	Receiving hospital code/name *(Core)*	*Note:* Copy of codes/ names used within each service to be provided separately Discharged to home or a lesser rehabilitation centre
	Survival to discharge *(Core)*	*Note:* Copy of codes/ names used within each service to be provided separately Discharged to home or a lesser rehabilitation centre Was the patient alive after 30 days?
	30 day survival *(Core)*	Did the patient have ROSC at point of arrival at the emergency department of the receiving hospital?
	Survival status (12 month) *(Supplemental)*	The patient is alive at 12 months after cardiac arrest
	Date of death *(Core)*	Date of death regardless of who confirmed *Note:* If ‘DNAR’ or ‘ROLE’ is ‘1 Yes’, then ‘Date of incident’ will replace ‘Blank’
	Date discharged *(Core)*	Date of discharge to home or a lesser rehabilitation centre
Process	Dispatcher identified presence of cardiac arrest *(Core)*	Did the ‘**Call Taker’** identify the presence of cardiac arrest before arrival of EMS? *Note:* if ‘Computer Aided Dispatch classification’ is ‘1 Red 1’, than ‘Blank, Unobtainable and Unknown’ will be overwritten as ‘1 Yes’
	Dispatcher provide CPR instructions *(Core)*	Did the ‘**Call taker**’ provide telephone CPR instructions to the caller? *Note:* if ‘Computer Aided Dispatch classification’ is ‘1 Red 1’, than ‘Blank, Unobtainable and Unknown’ will be overwritten as ‘1 Yes’
	Reported time of collapse at location *(Supplemental)*	What was the estimated time of collapse at the location of the incident if not witnessed by the person making the call? What was the estimated time of the witnessed collapse by either bystander or EMS
	Time of witnessed cardiac arrest by bystander or EMS *(Supplemental)*	Did the ‘**Call taker**’ provide telephone CPR instructions to the caller? *Note:* if ‘Computer Aided Dispatch classification’ is ‘1 Red 1’, than ‘Blank, Unobtainable and Unknown’ will be overwritten as ‘1 Yes’
	Time emergency medical services mobile *(Supplemental)*	The time the crew or individual responder is mobile following allocation of the incident The time the first emergency response vehicle stops at a point closest to the patient's location
	Time emergency medical services vehicle stops *(Core)*	*Note:* Copy of codes/ names used within each service to be provided separately Discharged to home or a lesser rehabilitation centre Was the patient alive after 30 days?
	Estimated time emergency medical services at patient’s side *(Supplemental)*	The moment of arrival at the patient’s side
Process continued	Defibrillation shock Time *(Core)*	Time of the first shock should be sourced from the external defibrillator clock regardless of initial source
	Estimated Defibrillation shock Time *(Supplemental)*	Best estimated time of the first shock regardless of initial source
	Defibrillation time *(Core)*	The time interval from incoming call (‘Call Connect time’) to the time the first shock is delivered
	Time of ROSC *(Core)*	Estimated time when the patient was noted to have a brief (approximately >30 s) restoration of spontaneous circulation that provides evidence of more than an occasional gasp, occasional fleeting palpable pulse, or arterial waveform
Not currently available to EMS	Independent living *(Supplemental)*	Before the cardiac arrest, the patient was able to perform all activities of daily living without the assistance of caregivers
	Comorbidities *(Supplemental)*	The patient has a documented history of other disease conditions that existed before the cardiac arrest
	Ventricular assist device *(Supplemental)*	The patient is supported by any form of ventricular assist device to augment cardiac output and coronary perfusion
	Cardioverter-defibrillation in place *(Supplemental)*	The patient has an internal or external cardioverter-defibrillator The time and setting where targeted temperature control was initiated
	Targeted temperature control *(C)*	Date of discharge to home or a lesser rehabilitation centre
	Targeted oxygenation/ ventilation *(Supplemental)*	After ROSC, was targeted ventilation applied?
	Reperfusion attempted *(Core)*	Was coronary reperfusion attempted?
	Extracorporeal life support *(Supplemental)*	When was extracorporeal life support used?
	Intra-aortic balloon pump *(Supplemental)*	Was an Intra-aortic balloon pump used?
	pH *(Supplemental)*	What was the first pH recorded after ROSC?
	Lactate *(Supplemental)*	What was the first lactate recorded after ROSC?
	Glucose *(Supplemental)*	After ROSC, was glucose titrated to a specific target?
	Neuroprognastication *(Supplemental)*	Number and type of neuroprognostic tests used
	Hospital type *(Supplemental)*	Was the patient's primary transfer to a healthcare facility able to perform all forms of periarrest and postarrest care and allocated this role by the area of administration?
	Hospital volume *(Supplemental)*	How many cases of OHCA does the hospital treat each year?
Not currently available to EMS	Targeted blood pressure management	What target blood pressure was used?
	Neurological outcome at hospital discharge *(Core)*	Record CPC and/or mRS or paediatric equivalent at hospital discharge. Include a definition of how it was measured (face to face, extracted from notes, combination)
	Survival status (12 month) *(Supplemental)*	The patient is alive at 12 months after cardiac arrest
	Treatment withdrawn *(Supplemental)*	A decision to withdraw active treatment was made. Record the time that this occurred after ROSC
	Cause of death *(Supplemental)*	Cause of death as officially recorded in the patient's medical records or death certificate The number of patients who had 1 or more solid organs donated for transplantation
	Organ donation *(Supplemental)*	The patient is alive at 12 months after cardiac arrest
	Patient reported outcome measures *(Supplemental)*	Patient-focused health outcomes were assessed
	Quality of life measurements	A validated quality-of-life measure was used to assess health quality of life

AED, Automated External Defibrillator; AHA, American Heart Association; ALS, Advanced Life Support; AQI, Ambulance Quality Indicator guidance; BLS, Basic Life Support; CAD, Computer Assisted Dispatch; CCCPR, Chest compression only CPR; CCG, Clinical Commissioning Group; CFR, Community First Responder; CPC, Cerebral Performance Score; CPR, Cardio Pulmonary Resuscitation; DNAR, Do Not Attempt Resuscitation order; EMS, Emergency Medical Services; EuReCa, European Registry of Cardiac Arrest; GP, General Practitioner; GI, Gastro Intestinal; HSCIC, Health and Social Care Information centre; ILS, Intermediate Life Support; mRS, Modified Rankin Scale; OforNS, Office for National Statistics; OHCAO, Out of Hospital Cardiac Arrest project; ROLE, Recognition of Life Extinct; SCR, Summary Care Record; STEMI, ST elevation in Myocardial Infarction.

Cardiac arrest event rate, patient characteristics, setting, clinical variables, process variables and outcomes will be presented using descriptive statistics. We will use multiple logistic regression models to examine the effect of prognostic factors on binary outcomes such as ROSC on arrival at hospital and survival to hospital discharge. The Kaplan-Meier or Cox regression model will be used to identify factors that may predict patient survival.

### Detailed study description

The study will be split into three phases: (1) initial feasibility, (2) data collection and (3) analysis and reporting.

#### Phase 1: initial feasibility

We will survey all 12 UK ambulance services to establish which patient, process and outcome variables, relevant to cardiac arrest are collected, how they are stored and what the data security systems are for each ambulance service. The feasibility questionnaire will be followed up by a one-to-one conversation with the identified lead to ensure data completeness and to seek clarification of any areas of uncertainty. We will request copies of existing data dictionaries related to cardiac arrest variables where these exist.

We will request anonymous samples of key cardiac arrest variables from ambulance services where these exist in electronic format which will be securely transferred to the Coordinating Centre.

These data will be used to produce a map of current processes for case identification, outcome verification and measurement/reporting of key cardiac arrest variables. We will explore the feasibility of changing to a unified approach of data management processes within UK ambulance services.

We will present the output from these surveys to the Steering Committee (SC) who will endorse which core and supplementary outcome variables will be recommended for collection in the main study.

Core variables will be prioritised based on importance and feasibility of data collection. Core variable selection will be informed by the Utstein recommendations for OHCA reporting^8^ ^31^ and will capture case mix, structure, process and outcomes.

#### Phase 2: data collection

##### Screening for eligibility

Case records of patients with suspected cardiac arrest will be identified by ambulance service personnel through the following screening processes:

- Search case records for clinical or treatment variables that are likely to occur in cardiac arrest, for example, zero pulse/zero respiratory, defibrillation
- Search case records for cardiac arrest
- Search 999 call database/dispatch systems for cardiac arrest dispatch codes

During the conduct of the project we work to achieve a standardised process for case identification.

##### Enrolment

Inclusion criteria:

1. Out of hospital cardiac arrest
2. Resuscitation is attempted (Advanced or Basic Life Support) commenced/continued by ambulance service

Exclusion criteria:

1. Arrest during inter-hospital transfer or on acute NHS hospital trust premises
2. Clear evidence of death defined by the Joint Royal College Ambulance Liaison Committee (JRCALC)^17^ recognition of life extinct (ROLE) criteria. See online supplementary data for these criteria

##### Variables being collected

Core and supplemental variables that will be collected will cover the following headings:

- Patient identifiable information
- Patient characteristics
- Event data/clinical information
- EMS response variables/interventions
- Outcome variables

### Database

The Out of Hospital Cardiac Arrest Outcome (OHCAO) registry system is an Extract Transform Load (ETL) web application and database for aggregating and processing data obtained from the UK’s Ambulance Services. The set up and management of this database will also comply with Warwick Clinical Trials Unit (WCTU) Standard Operating Procedures (SOPs) on data security and data management and the University of Warwick's data security policy. The system comprises a SQL Server database for storing data obtained from each ambulance service and an ASP.NET web application hosted on an IIS 6 web server. An additional SQL Server database is used to host a replicated copy of the registry for analysis and reporting. SQL Server Reporting Services (SSRS) is used for all reporting requirements. The web application prohibits users from viewing the import history from other ambulance services.

### Determining patient outcomes

Resuscitation is terminated at the scene of the cardiac arrest in approximately 30% of cases.^32^ The remaining 70% are transferred to hospital of which approximately two-thirds have resuscitation efforts terminated in the emergency department.^32^ Of those patients who initially survive and are admitted to hospital, only approximately half survive to go home. Tracking these patients to determine their outcome is complex and time consuming because it involves manual follow-up from the 14 ambulance services with over 220 acute NHS Trusts. We propose to explore the possibility to standardise and streamline the process for outcome verification for those patients who did not die in the care of the ambulance services, to determine whether or not these survivors died subsequently (and if so, why and when).

We will attempt to match patients who are known by the ambulance service to obtain a ROSC with data held by the Health and Social Care Information Centre (HSCIC). We will also sample 10% of patients across all ambulance services where resuscitation is attempted. We will utilise the HSCIC Data Linkage and Extract Service to establish survival status of these patients at 30 days following cardiac arrest. We will use the flagging service to follow long-term survival. We will measure the proportion of patients where it is possible to obtain a match and compare 30 day survival status with the survival to hospital discharge information provided by the ambulance service.

Once a match has been obtained with HSCIC, we will delete non-essential patient identifiable information, retaining only the study unique ID to allow later updating of death status. Patient's NHS number, date of birth and postcode will also be retained to allow future data linkage for further assessment of sources of variation (ie, intensive care management, cardiovascular interventions) that influence survival rates.

### Ethical considerations

We have carefully considered the data that are required to examine the epidemiology and outcome of OHCA. Ethics committee permissions were gained from South Central—Oxford C Research Ethics Committee (reference 13/SC/0361). The study also has received approval from the Confidentiality Advisory Group (CAG) Ethics and Confidentiality committee (ECC 8-04(C)/2013), which provides authorisation, on behalf of the Secretary of State, to lawfully hold identifiable data on patients without their consent. We will comply with the common law duty of confidentiality owed by health professionals in regard to information provided by patients in the course of clinical care, and the principles of the Data Protection Act 1998, which apply to the processing of data by Research Databases in the same way as to specific research projects. The project has received approval from the CAG for permission to implement Section 251 of the NHS Act 2006 (originally enacted under Section 60 of the Health and Social Care Act 2001), which allows identifiable patient information to be used without consent in very specific circumstances. The CAG approval also provides the SC with the authority to provide other researchers access to anonymised data in specific circumstances.

#### Phase 3: analysis and reporting

We anticipate having data on at least 35 000 cardiac arrests by the end of the project. The study statistician will develop and present a detailed statistical analysis plan to the Steering Committee for approval prior to data analysis.

We anticipate using descriptive statistics to summarise patient characteristics, clinical variables, EMS dispatch characteristics, EMS process variables, location and cardiac arrest event rate. Data will be presented for the entire population, the Utstein comparator group (witnessed arrest, bystander CPR, shockable rhythm) and broken down by ambulance service.

We are interested in the outcomes of ROSC and patient survival to hospital discharge. Both outcomes are binary variables where in the latter variable, the dichotomy is whether the patient survives to be discharged from hospital or not.

Potential factors that may explain the binary outcome will be identified using logistic regression model with ambulance services as random effects. Factors that have been identified will be included in a multiple logistic regression model with ambulance services as random effects to assess their inclusion in the risk prediction model.

We will also explore which factor may predict patient survival with either the Kaplan-Meier or Cox regression model. Factors that are relevant will be investigated further in a multiple Cox regression model. Both univariate and multivariate modelling will be adjusted by ambulance services. Survivors at time of analysis will be treated as censored cases.

However, as some prognostic factors may be correlated, we will assess multicollinearity to avoid including prognostic factors that are highly correlated in the same model.

As submitting data to the database is not compulsory, and there is a large variability in data quality of individual patient data, the data may be incomplete because of missing observations for the outcomes or the prognostic factor. This was previously the experience of other databases such as MINAP (Myocardial Ischaemia National Audit Project). Missing data may also follow some pattern, which would lead to biased results if appropriate methods are not used.

### Quality improvement reports

The main risk prediction modelling will be based on complete case analysis. We will assess the pattern of missing data and consider multiple imputation.

In collaboration with the Steering Committee and collaborating ambulance services we will agree on the content of reports that will be provided for ambulance services. It is envisaged that initial reports will focus on data completeness, timeliness and quality. Subsequent reports will summarise demographic, patient, process and outcome variables. It is anticipated that data will be presented in summary form and broken down by ambulance service. Identification of ambulance service in any reports will be by unique code (known only to the ambulance service concerned). Reports will be sent to the principle investigator at each ambulance service and members of the Steering Committee.

## Summary

Improving patient outcomes from OHCA is a key priority for the NHS. To identify the key characteristics contributing to better outcomes in some ambulance services, reliable and reproducible systems need to be established for collecting data on OHCA in the UK. The aim of this project is to establish the epidemiology and outcome of out of hospital cardiac arrest, explore sources of variation in outcome and establish the feasibility of setting up a national OHCA registry.
